# Patient use of a self‐monitoring app during eating disorder treatment: Naturalistic longitudinal cohort study

**DOI:** 10.1002/brb3.2039

**Published:** 2021-01-18

**Authors:** Pil Lindgreen, Kirsten Lomborg, Loa Clausen

**Affiliations:** ^1^ Department of Child and Adolescent Psychiatry Aarhus University Hospital Aarhus Denmark; ^2^ Clinical Research Steno Diabetes Center Copenhagen Gentofte Denmark; ^3^ Department of Clinical Medicine Faculty of Health Aarhus University Aarhus Denmark

**Keywords:** eating disorders; smartphone; anorexia; bulimia

## Abstract

**Objective:**

To explore patients’ use of the self‐monitoring app Recovery Record during 26 weeks of naturalistic eating disorder treatment.

**Methods:**

Selected patient characteristics at baseline were explored as predictors of app use using linear regression. Patients were grouped according to diagnosis (anorexia versus bulimia), and mixed‐effects analyses were used to explore differences in app use between diagnoses across four time periods (weeks 1–4; weeks 5–8; weeks 9–12; weeks 13–26).

**Results:**

Eighty‐four patients were included of which 41 had anorexia and 43 had bulimia. The total number of logs varied greatly (mean (*SD*): 592 (628.50)), and patient app activity almost ceased at week 13. Increasing age and no previous eating disorder treatment predicted increased app activity (*p* = .007; *p* = .039, respectively). Patients with anorexia logged over four times more often than patients with bulimia in the last time period (median (CI): 4.27 (1.28;14.31); *p* = .018). Time predicted declining app use (all *p* ≤ .007).

**Conclusion:**

Future research on long‐term app engagement should investigate associations between patients’ app use and changes in their eating disorder symptom severity over time.

## Graphical abstract

The study aimed to explore 84 patients’ use of a meal self‐monitoring app during 26 weeks of naturalistic eating disorder treatment. Patient app activity varied greatly, and by week 13, most patients did not use the app anymore. Increasing age and no previous eating disorder treatment predicted increased app activity, and patients with anorexia logged over four times more often than patients with bulimia by the end of the studied time period. Future research should investigate associations between patients’ app use and their type and severity of eating disorder symptoms over time.

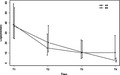

## 1 SIGNIFICANT OUTCOMES

- Patient app activity varied greatly in the studied eating disorder treatment setting.
- Eating disorder diagnoses did not predict patients’ long‐term app engagement, but age, previous eating disorder treatment, and time may be relevant predictors.

## 2 LIMITATIONS

- The study is neither controlled nor randomized.
- Large confidence intervals for total number of logs suggest a lack of statistical power.
- The study applies a median split approach to define “high” and “low” app activity groups which may conceal individual‐level variation in the data.

## 3 INTRODUCTION

The field of mobile health is rapidly growing, and in 2017, there were more than 325,000 health‐related software applications (apps) available globally (Research2guidance, 2017). The effectiveness of many mental health‐related apps as digital treatment add‐ons has been established (Linardon et al., 2019). Still, knowledge gaps remain, for instance on the impact of app‐delivered services on the perception of self (Lupton, 2013a), and the therapeutic alliance between patients and healthcare professionals (Fairburn & Patel, 2017; Fairburn & Rothwell, 2015). The use of mental health apps may provide more people, attending established treatment or not, with the support that they need by breaking down geographical and financial barriers to treatment (Van Ameringen et al., 2017) as well as more novel barriers occurring due to restrictions following the COVID‐19 pandemic (Weissman et al., 2020). Furthermore, apps allow for several interactive, innovative technical features that may improve treatment adherence and outcomes, such as nudging (i.e., the prompting of end‐users to perform a behavior), gamification (i.e., incorporating game‐like aspects into nongame settings), and in‐app clinician messaging (Basten, 2013, 2017; Linardon et al., 2019; Lupton, 2014; Malvey & Slovensky, 2014; Vlaev et al., 2016). Although apps do not currently undergo any systematic quality control regarding their validity, evidence‐base, or theoretical soundness before being launched (Lupton, 2014), apps are becoming increasingly attractive to clinicians as part of blended treatment (i.e. the mixture of digital and face‐to‐face treatment; Lupton, 2013b).

In eating disorder (ED) treatment, many patients are young and therefore likely to be familiar with and respond well to apps (Donovan, 2016). Additionally, previous studies have documented patient‐reported benefits of apps in ED management; besides convenience, patients have been shown to appreciate the option to adjust the explored apps to fit their individual needs along with the in‐app social support provided by peers and clinicians (Basterfield et al., 2018; Juarascio, Goldstein, et al., 2015; Nitsch et al., 2016). One commonly used app in ED treatment is Recovery Record (RR), which is primarily based on cognitive behavioral therapy (CBT) (Juarascio, Manasse, et al., 2015). RR can be used for self‐management or in ED treatment, where clinicians can monitor patients’ app data after linking with them via the app (Tregarthen et al., 2015).

Similar to traditional CBT‐based pen‐and‐paper meal diaries, the purpose of RR is for patients to digitally self‐monitor their meals, physical state, emotions, thoughts, and behaviors (Fairburn et al., 2003). To increase self‐monitoring adherence (Juarascio, Manasse, et al., 2015), RR encompasses meal and logging reminders, postlogging affirmations, and gamification features (Tregarthen et al., 2015). Still, a recent study comparing RR with paper meal diaries applied in outpatient ED treatment did not detect statistically significant differences neither on acceptance, adherence levels, nor eating disorder symptomatology over time (Keshen et al., 2019). Another study, however, found high levels of acceptance of RR overall in patients following inpatient treatment for anorexia nervosa (AN), when compared to patients following usual aftercare (Neumayr et al., 2019). Patients found the feature of linking with their clinician to be the most helpful in overcoming their ED symptoms during aftercare (Neumayr et al., 2019).

Long‐term user engagement, which can be defined as a user's degree of involvement in a digital system over time (Bickmore et al., 2010), is typically expected to decrease steadily in users of health‐related apps (Bickmore et al., 2010; O'Connell, 2016; Taki et al., 2017). In conventional ED treatment, attrition, which can be defined as the rate of patients dropping out of treatment altogether (Fassino et al., 2009), is a general issue with dropout rates as high as 73 percent for outpatients (Fassino et al., 2009). Treatment dropout is associated with poorer outcomes, such as increased binging and purging behavior in people with bulimia nervosa (BN) and lower body mass index values in people with AN (Vall & Wade, 2015). Thus, although concerning, high drop‐out rates are expected in ED treatment, but little is known about patients’ long‐term engagement in apps applied as part of ED treatment. A recent longitudinal study explored the changes in ED symptoms in RR users during three months (Chapa et al., 2019). The study found major significant improvements on most ED symptomatology when assessed by the Eating Pathology Symptoms Inventory (Forbush et al., 2013) measuring body dissatisfaction, binge‐eating, cognitive restraint, purging, restricting, and excessive exercising (Chapa et al., 2019). Still, the study did not report on the participants’ app engagement, such as the change in the number of completed logs over time. Our longitudinal cohort study may therefore provide novel insights on the long‐term app engagement of outpatients in ED treatment.

Due to methodological issues (including study dropout) in and across studies, more knowledge on reasons for treatment dropout is warranted to guide the design of future treatment interventions (Fassino et al., 2009), including digital ones. Among suggested reasons for decreasing long‐tern engagement in ED treatment apps are limited support from therapists, difficulties navigating in the applied app, and unpleasant feelings when logging meals or symptoms (e.g., feelings of shame when logging and revisiting the amount of food eaten or when logging binging or purging behaviors) (Basterfield et al., 2018; Lindgreen, Lomborg, et al., 2018). However, to the best of our knowledge, no studies have explored predictors of app use at ED treatment baseline. ED diagnosis may be a predictor of patient app use due to differences in core ED symptoms (e.g., binge‐eating in BN versus dietary restriction in AN) and ED treatment goals (e.g., weight stabilization in BN versus weight gain in AN) (Accurso et al., 2014; National Institute for Health & Care Excellence, 2017). For instance, logs on feelings in relation to binging behavior will likely be more applied in patients with BN than in those with AN, which will likely affect how the app is used. Personality traits, which are also linked to ED diagnosis, may also affect patient app use; although both AN and BN are consistently associated with perfectionism, obsessive‐compulsiveness, and neuroticism, AN has been linked to higher levels of constraint, persistence, and obsessive‐compulsiveness than BN, whereas BN has been associated with higher impulsivity and sensation seeking than AN (Cassin & Ranson, 2005). These differences in personality traits may potentially result in differing app use in patients with AN compared with BN, for instance by resulting in regular and meticulous app engagement in AN as opposed to an irregular or fluctuating app use in BN. As higher illness severity, including prolonged illness duration, is associated with lower levels of motivation to recover (Vall & Wade, 2015) and less beneficial outcomes of ED treatment (Cooper et al., 2016), illness severity may also be a predictor of app engagement to be investigated. Previous studies have found poor baseline interpersonal functioning, which can be defined as problems related to a person's social interactions and engagement with others (Hartmann et al., 2009), to predict worsened outcomes over time in patients with AN or BN (Vall & Wade, 2015). Thus, interpersonal functioning may influence patients’ use of an app as part of treatment. Finally, younger age may predict high app engagement in patients, as younger patients are likely to be more familiar and comfortable with apps in general than older patients (Donovan, 2016). Thus, we wanted to explore patients’ long‐term engagement in RR as part of ED treatment along with the association between patients’ app engagement and baseline characteristics.

### 3.1 Aims of the study

The first aim of the study was to explore the long‐term engagement in a self‐monitoring app applied in a naturalistic Danish patient sample by measuring the number of logs performed during the initial 26 treatment weeks. The second aim of the study was to explore potential predictors of app use by investigating associations between patient app usage and selected patient characteristics at baseline.

## 4 MATERIAL AND METHODS

### 4.1 Setting and sample

The study took place at a Danish two‐centered ED clinic receiving around 650 annual referrals of patients with moderate to severe EDs. The clinic employs approximately 60 clinicians working in multidisciplinary teams consisting of psychiatrists, psychologists, dietitians, nurses, physiotherapists, and occupational therapists, all with a minimum of a bachelor's degree. Outpatient treatment is offered for (atypical) AN and BN, whereas inpatient treatment is only offered for AN. Initially, patients undergo a clinical assessment, where their psychological, social, and somatic history is appraised, a medical examination is performed, and a patient questionnaire on socio‐demographic information and the Eating Disorder Inventory 2 (EDI‐2) are completed. ED diagnoses are determined using the semi‐structured Eating Disorder Examination (EDE) (Fairburn et al., 2008). Treatment plans are decided at multidisciplinary conferences and depend on the individual patient's ED diagnosis, age, and living situation (e.g., with parents). Standard treatment for adults with AN includes weekly therapy alternating between individual and group sessions, the latter including group therapy and a clinician‐supported joint meal. The individual sessions emphasize the normalization of weight and eating using elements from CBT, specialized supportive clinical management, and interpersonal therapy (IPT) (McIntosh, 2015; Murphy et al., 2012; National Institute for Health & Care Excellence, 2017). Adolescents with AN are offered the same treatment combined with family‐based treatment (FBT) instead of IPT, or only FBT (Lock & Le Grange, 2015). For BN, adolescents and young adults living with their parents are offered weekly group CBT supplemented with a clinician‐supported joint meal with their parents every other week (Hollesen et al., 2016). The standard treatment for adults with BN is 10 weekly group sessions (Davis, & Olmsted, 1992; Jones & Clausen, 2013), that is, if needed, followed by additional group or individual therapy. Adult nonresponders are offered day hospital treatment consisting of three week‐day sessions for four months. Some patients are offered individually tailored treatment if they are unlikely to benefit from group therapy (e.g., due to severe comorbidity). When conducting the study, binge‐eating disorder was not treated at the clinic and therefor not included in the study. Patients in FBT were also excluded, as patient self‐monitoring does not match its rationale (Lock & Le Grange, 2015).

All clinicians received the RR handbook, participated in two one‐hour RR group training sessions, and were encouraged to ask for additional support if needed. The RR handbook was translated from English in agreement with RR Inc. by the first author, who also provided the clinician training and additional support. In patients’ initial treatment phase, clinicians discussed the rationale of self‐monitoring with patients and encouraged them to self‐monitor using pen‐and‐paper or via RR. As part of the naturalistic treatment setting, the individual patients and clinicians collaborated on deciding the proper self‐monitoring tool along with how and when to incorporate the RR data in‐session. Patients were allowed to use all features of the app, except for the direct messaging feature, as it was not allowed in Denmark at the time for legal reasons (Danish Ministry of Finance, 2016).

For 18 months starting 1 February 2016, we recruited outpatients with AN or BN (according to the 5th edition of the Diagnostics and Statistical Manual of Mental Disorders (DSM‐5) (American Psychiatric Association, 2013), with a lower age‐limit of 15 years. Inpatients and patients with psychotic or developmental disorders were excluded. Following the assessment, eligible patients were invited to participate by their primary clinician or the first author. Accepting patients and, where relevant, their legal guardian(s) signed an informed consent form. The study was approved by the Danish Data Protection Agency (ID: 1–16–02–313–15).

### 4.2 Data material

Socio‐demographic data (i.e., age; occupation; living situation; relationship status; previous ED treatment) were collected from a study‐specific questionnaire.

Clinical data were collected from medical records at baseline (i.e., EDE and EDI‐2; psychiatric comorbidity; treatment type) and at end‐of‐treatment (treatment duration; number of inpatient admissions). During the study, the EDE Questionnaire (EDE‐Q) (Fairburn & Beglin, 2008) was issued digitally every five weeks, but despite automatic reminders, the response rate was low (48.8% at first and 23.8% at final follow‐up). Thus, the data were omitted due to the lack of statistical power.

App data included the date of patient–clinician linking and the individual patient's daily number of various log types: 1) meals; 2) meal photographs/thoughts/feelings; 3) restriction; 4) exercise; 5) skipped meals; 6) other ED behavior (e.g., weighing); 7) urges (e.g., to binge); 8) binging; 9) purging; 10) use of diet pills; 11) use of laxatives; 12) applied coping strategies; and 13) applied goal setting. The initial seven log types were included, while the remaining were excluded due to lack of use (≤5 logs for each log type across all patients during the 26 study weeks). Post hoc, a brief e‐mail survey on reasons for not using RR was issued to patients who never engaged with it (*n* = 18). All data, except for data from medical records, were collected specifically for the purpose of this study.

### 4.3 Hypotheses

We hypothesized that increased app activity would be predicted by a diagnosis of AN (compared with BN); decreasing age; being in a relationship (as a measure of interpersonal functioning); and no previous ED treatment (as a measure of illness severity).

### 4.4 Statistical analyses

The main outcome variable was patients’ weekly app activity level. To compare high versus low app activity levels, the sample was divided into two app activity groups (“high” versus “low”) using the median split approach (median = 3.6 “active” weeks) (Iacobucci et al., 2015a, 2015b). This approach was applied due to the limited sample size and to avoid the loss of statistical power (Iacobucci et al., 2015a, 2015b). Patient baseline characteristics were compared between these two app activity groups and between diagnostic groups (AN versus BN). To account for individual patient variance in daily app use during each week, four app activity categories based on weekly activity levels were defined:

1. “Inactive”: 0 logs per week.
2. “Low activity”: 1 day of logging (≥ 1 log/day) per week.
3. “Moderate activity”: 2–4 days of logging per week (≥ 1 log/day).
4. “High activity”: 5–7 days of logging per week (≥ 1 log/day).

Categories 3 and 4 were collapsed into one “active” category to calculate each patient's number of “active” app weeks.

To investigate whether diagnosis predicted the number of “active” app weeks, linear regression was performed while the impact of age, relationship status, and previous ED treatment was also tested.

Data were logarithmically transformed before running the mixed‐effects analyses due to skewedness (Lindgreen, Lomborg, et al., 2018). Mixed‐effects analysis was performed to explore the interaction between diagnosis and time, along with the impact of this interaction on app use. Thus, we calculated the mean number of all logs for four time periods (i.e. weeks 1–4 (T1); weeks 5–8 (T2); weeks 9–12 (T3); weeks 13–26 (T4)). T4 was defined as a longer time period due to the sparse app activity late in treatment. In the model, diagnosis, time, and their interaction were included as fixed effects, patients as a random effect, and previous ED treatment and relationship status, which we controlled for, as fixed effects. It was tested whether the AN and the BN groups had parallel patterns of app use, and if their mean number of logs significantly differed at the four time points.

Because the study was exploratory, post hoc mixed‐effects analyses were conducted to investigate the impact on app use of the interactions between i) time and age (as a binary variable defined using the median split approach (Iacobucci et al., 2015b) (median = 21.5 years)) while controlling for previous ED treatment (yes/no) and relationship status (single/in a relationship), and ii) time and previous ED treatment while controlling for age and relationship status. Previous ED treatment and age were selected for post hoc analyses because they were significant predictors of app use in the linear regression analysis. The survey sent to nonusers was analyzed descriptively. All statistical analyses were conducted using Stata 15® (Stata Nordic, 2018).

## 5 RESULTS

### 5.1 Baseline characteristics

Initially, 90 patients agreed to participate, but six (6.7%) dropped out, leaving 84 patients (41 with AN; 43 with BN) (Table 1). Six patients had an “other specified feeding or ED,” but were grouped as AN, as the combination of their ED behavior, psychopathology, and body mass index resembled an anorexic type ED more than a bulimic one. Socio‐demographic data were collected for all 84 patients, EDE data for 79 patients (94.0%; 28 with AN and 41 with BN), and EDI‐2 data for 51 patients (60.7%; 27 with AN and 24 with BN). As expected, significant differences were found between patients with AN versus BN on key diagnostic criteria (i.e. body mass index; binging and purging behavior (EDE); shape concern (EDE subscale); and bulimia scale (EDI‐2)) (Table 1). No other differences were detected.

**TABLE 1:** Baseline characteristics for patients with AN versus BN (*N* = 84)

	AN	BN	*p*^d^
	*N* (%)	*N* (%)	
Patients	41 (48.19)	43 (51.81)	
Age	Mean (*SD*)	Mean (*SD*)	.774
Years	22.61 (5.60) (range: 15–41)	22.42 (4.81) (range: 15–36)	
Grouped age (years)	*N* (%)	*N* (%)	
15–20	16 (39.02)	16 (37.21)	
21–25	17 (41.46)	18 (41.86)	
26–30	5 (12.20)	5 (11.63)	
≥31	3 (7.32)	4 (9.30)	
BMI	Mean (*SD*)	Mean (*SD*)	**.001**
	17.49 (1.73) (range: 14.32–21.48)	22.03 (2.04) (range: 18.16–27.40)	
Grouped BMI	*N* (%)	*N* (%)	
<15.0	3 (7.32)	0 (0.00)	
15.0–18.4	27 (65.85)	1 (2.33)	
18.5–19.9	6 (14.63)	6 (13.95)	
20.0–24.9	5 (12.20)	34 (79.07)	
≥25.00	0 (0.00)	2 (4.65)	
EDE scores	Mean (*SD*)	Mean (*SD*)	
Restraint	3.79 (1.12) (*n* = 38; range: 0–5.40)	3.67 (1.46) (*n* = 43; range: 0–6)	.872
Eating concern	2.79 (1.46) (*n* = 38; range: 0–5.20)	3.20 (1.29) (*n* = 42; range: 0–6)	.267
Shape concern	3.56 (1.66) (*n* = 38; range: 0.21–5.93)	4.34 (1.39) (*n* = 41; range: 1.14–6)	**.023**
Weight concern	3.42 (1.78) (*n* = 38; range: 0–6)	4.13 (1.66) (*n* = 41; range: 0.50–6)	.066
Total	3.39 (1.25) (*n* = 38; range: 0.05–5.20)	3.88 (1.20) (*n* = 41; range: 1.52–5.76)	.137
EDE scores (episodes/week)	Mean (*SD*)	Mean (*SD*)	
Obsessive exercise	4.94 (2.00) (*n* = 25; range: 0–7)	4.60 (2.03) (*n* = 25; range: 0–7)	.519
Binging	2.75 (7.93) (*n* = 22; range: 0–	6.28 (6.26) (*n* = 37; range: 0–	**.001**
Purging^e^	35) 3.36 (7.53) (*n* = 21; range: 0.08–35)	28) 8.67 (10.09) (*n* = 35; range: 0.08–50)	**.001**
Treatment	*N* (%)	*N* (%)	
Standard AN/BN^f^	32 (80.49)	37 (86.05)	
Individual AN/BN^g^	6 (14.63)	6 (13.95)	
Day hospital	2 (4.88)	0 (0.00)	
Treatment length (weeks)	Mean (*SD*)	Mean (*SD*)	.817
	24.16 (4.46) (range: 5.29–26.00)	23.39 (5.25) (range: 3.71–26.00)	
Inpatient treatment	*N* (%)	*N* (%)	
Yes (during study)	8 (19.51)	0 (0.00)	
Previous EDtreatment (no.)^a^	*N* (%)	*N* (%)	
Yes	16 (39.00) (range: 0–8) ^a^	22 (51.16) (range: 0–8) ^a^	.264
1–2	13 (31.71)	16 (37.21)	
≥3	3 (7.32)	6 (13.95)	
Psychiatric comorbidity^b^	*N* (%)	*N* (%)	.269
Yes	16 (39.02)	13 (30.23)	
Anxiety/OCD	4 (9.76)	7 (16.28)	
Depression	10 (24.39)	7 (16.28)	
Personality disorder	2 (4.88)	4 (9.30)	
Other	1 (2.44)	6 (13.95)	
Occupation^c^	*N* (%)	*N* (%)	.067
Employee/student	30 (73.17)	38 (88.37)	
Unemployed	3 (7.32)	1 (2.33)	
Sick leave	8 (19.51)	2 (4.65)	
Other	0 (0.00)	2 (4.65)	
Relationship status	*N* (%)	*N* (%)	.791
Single	25 (60.98)	25 (58.14)	
In a relationship/married	16 (39.02)	18 (41.86)	
Living situation	*N* (%)	*N* (%)	.510
Alone	21 (51.22)	26 (60.47)	
With parents/partner	20 (48.78)	17 (39.53)	

Note

Abbreviations: AN, anorexia nervosa; BMI, body mass index; BN, bulimia nervosa; ED, eating disorder; EDE, ED Examination; OCD, obsessive compulsive disorder; *SD*, standard deviation.

^a^ The number of previous courses of ED treatment.

^b^ "Other" includes post‐traumatic stress disorder and attention‐deficit hyperactivity disorder. Psychiatric diagnoses were made by psychiatrists and/or clinical psychologists using neurodevelopmental assessment tools (). Some patients (AN: *n* = 2; BN: *n* = 5) had several diagnoses.

^c^ "Other" includes maternity leave or job training.

^d^ *t* test for parametric continuous variables; Mann–Whitney U test for continuous nonparametric variables; chi‐squared test for categorical variables.

^e^ As no patients used diuretics and only seven (AN: *n* = 4; BN: *n* = 3) used laxatives, these two categories were collapsed with vomiting into one purging category.

^f^ See the methods section.

^g^ Refers to individually tailored treatment.

### 5.2 Frequency and duration of app use

During the 26 study weeks, 18 (21.4%) patients did not use the app (Table 2). No significant differences on baseline characteristics between app users and nonusers were detected. Of the 18 nonusers, 10 (55.6%) responded to the post hoc survey, primarily citing “not wanting to focus on eating” (*n* = 6), “lacking mental surplus” (*n* = 4), “forgetting” (*n* = 4), and “not wanting to log in the company of others” (*n* = 4) as reasons for not using the app.

**TABLE 2:** App data for patients with anorexia nervosa (AN) versus bulimia nervosa (BN) (*N* = 84)

	AN	BN	*p*^a^
	*N* (%)	*N* (%)	
Patients	41 (48.19)	43(51.81)	
Did not make an account	6 (14.63)	3(6.98)	.520
Made account but did not log	4 (9.76)	5(11.63)	
Number of logs^b^	Mean (*SD*)	Mean (*SD*)	
Meals	205.51 (225.80) (*n* = 31; range: 1–865)	186.15 (218.92) (*n* = 35; range: 2–877)	.675
Meal photographs/thoughts/feelings	251.34 (300.90) (*n* = 31; range: 1–1,186)	209.60 (243.37) (*n* = 35; range: 1–1,065)	.686
Other eating disordered behavior	37.94 (104.58) (*n* = 23; range: 1–565)	34.53 (71.34) (*n* = 22; range: 1–275)	.378
Restriction	34.46 (93.25) (*n* = 23; range: 1–491)	27.48 (66.85) (*n* = 30; range: 2–306)	.489
Meals skipped	7.94 (12.56) (*n* = 23; range: 1–43)	12.55 (23.30) (*n* = 23; range: 1–103)	.913
Exercise	1.83 (6.53) (*n* = 5; range: 1–29)	0.38(1.46) (*n* = 3; range: 2–7)	.336
Urges	8.86 (17.24) (*n* = 12; range: 1–55)	26.73 (49.51) (*n* = 19; range: 1–210)	.141
Total	547.89 (645.10) (*n* = 31; range: 2–2,588)	497.00 (604.63) (*n* = 35; range: 1–2,568)	.878
App delay (days)	Mean (*SD*)	Mean (*SD*)	
From assessment to linking	9.51 (13.76) (*n* = 35; range: 0–41)	5.86 (10.45) (*n* = 40; range: 0–41)	.158
From assessment to first log	10.56 (9.43) (*n* = 31; range: 0–48)	7.09 (7.62) (*n* = 35; range: 0–44)	**.003**

Note

Abbreviations: AN, anorexia nervosa; BN, bulimia nervosa; *SD*, standard deviation.

^a^ *t* test, Mann–Whitney U test, or chi‐squared test.

^b^ Accumulated over 26 weeks for active patients (*n* = 66).

The mean number of logs in patients using the app (*n* = 66) during the 26 initial treatment weeks was 592 (*SD* = 628.50, range: 2–2,588). The patients mostly recorded meal logs and meal photographs/thoughts/feelings, while rarely engaging with the remaining log types (Table 2). Patients with AN completed more logs than patients with BN, but the difference was not statistically significant. Yet, patients with AN started logging significantly later than patients with BN. The number of logs peaked in T1 and gradually decreased to less than six logs per week from week 13 (T4) (Table 3).

**TABLE 3:** Number of total logs over time according to diagnosis (anorexia nervosa (AN) versus bulimia nervosa (BN)) (*n* = 66)

ED diagnosis	T1 (weeks 1–4)	T2 (weeks 5–8)	T3 (weeks 9–12)	T4 (weeks 13–26)
	Mean (*SD*)	Mean (*SD*)	Mean (*SD*)	Mean (*SD*)
AN	46.03 (48.06) (range: 0–163.50)	29.87 (43.19) (range: 0–132.75)	21.73 (38.21) (range: 0–120.50)	5.51 (15.39) (range: 0–79.64)
BN	45.24 (46.63) (range: 0–152.50)	30.86 (36.68) (range: 0–118.25)	21.28 (35.96) (range: 0–142.25)	5.23 (14.26) (range: 0–65.43)
Total	45.63 (47.05) (range: 0–163.50)	30.38 (39.75) (range: 0–132.75)	21.51 (36.85) (range: 0–142.25)	5.36 (14.73) (range: 0–79.64)

Note

Abbreviations: AN, anorexia nervosa; BN, bulimia nervosa; ED, eating disorder; *SD*, standard deviation; T, time period.

### 5.3 Factors associated with app use

Patients in the “high” app activity group had a significantly higher score on the EDE restraint subscale than patients with “low” app activity (mean (*SD*): 4.02 (1.26) versus 3.44 (1.30); *p* = .046). No other statistically significant differences were detected between these groups.

The linear regression showed no significant association between ED diagnosis and the number of “active” weeks using RR (Table 4). In fact, although not significant, AN was associated with fewer “active” weeks than BN. Higher age was significantly associated with an increase in “active” weeks using RR, whereas previous ED treatment was significantly associated with a decrease in “active” weeks.

**TABLE 4:** Linear regression (number of weeks with active app use) (*N* = 84)

Variable	Coefficient	*SE*	*p*	95%CI
BN	Ref.			
AN	−0.93	1.24	.454	−3.41;1.54
Age (years)	0.35	0.13	**.007**	0.10;0.60
In relationship/married	Ref. −2.12	1.28	.101	−4.66;0.42
Single	Ref.			
No previous ED treatment	−2.73	1.30	**.039**	−5.31;−0.15
Previous ED treatment Constant	0.23	2.81	.935	−5.36;5.58
*R^2^* = .12				

Note

Abbreviations: AN, anorexia nervosa; BN, bulimia nervosa; CI, confidence interval; ED, eating disorder; SE, standard error.

**FIGURE 1:**
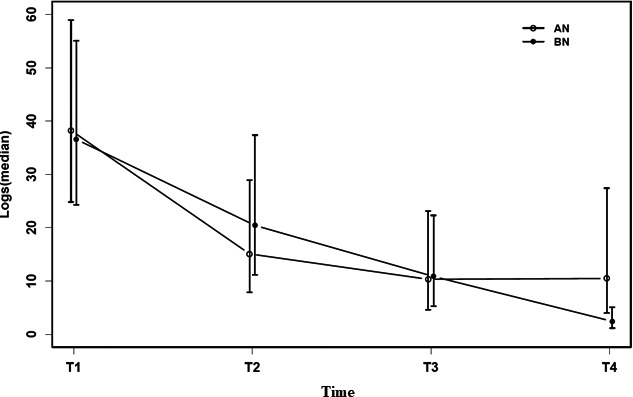
Patient app use over time according to diagnosis (anorexia nervosa (AN) versus bulimia nervosa (BN)) (*n* = 66). The time periods T1, T2, T3, and T4 refer to treatment weeks 1–4, 5–8, 9–12, and 13–26, respectively. Abbreviations: AN, anorexia nervosa; BN, bulimia nervosa

The mixed‐effects analysis of the mean number of logs revealed a parallel long‐term app engagement in the AN and BN groups, with no significant differences between the two (*p* = .058) (Figure 1). There was an interaction between diagnosis and time (when controlling for age, relationship status, and previous ED treatment) that differed significantly at T4, where the AN group recorded a quadruple amount of logs compared with the BN group (median (CI): 4.27 (1.28;14.31); *p* = .018). Time was in itself a significant predictor of declining patient app use (median ratios (CI): 0.56 (0.37;0.85), *p* < .007; 0.30 (0.16;0.54), *p* < .001; 0.07 (0.03;0.14), *p* < .001 for T2, T3, and T4, respectively, compared with T1).

The post hoc mixed‐effects analyses revealed no significant interaction between time and age or time and previous ED treatment. Only at T1, a difference was observed, with the older age group logging almost twice as much as the younger one (median ratio (CI): 1.94 (1.07;3.53); *p* = .029).

## 6 DISCUSSION

The number of patient‐recorded logs varied greatly, peaked within the first four treatment weeks, and then gradually decreased with the highest dropout occurring from week 13 (T4). This corresponds to previous research observing that engaging individuals in long‐term, web‐facilitated logging in health‐related settings is, in general, challenging (Bickmore et al., 2010; Neve et al., 2010; O'Connell, 2016; Taki et al., 2017). The substantial decrease in app engagement reported in our study might have been lower, however, if the patient‐clinician in‐app messaging feature had been permitted, as ongoing clinician support and feedback facilitated by online platforms may increase user engagement and retention (Neumayr et al., 2019; Neve et al., 2010). Similarly, in the study of RR as part of aftercare following inpatient treatment for AN, patients highly valued receiving continuous clinician feedback and the option to message their clinician directly (Neumayr et al., 2019). Thus, ED treatment clinics in countries, in which the direct messaging feature is permitted, should consider prioritizing the use of this feature to best accommodate patients’ needs. Although in‐app clinician support is much appreciated by app users, people with EDs who do not attend ED treatment may, according to previous research, also benefit from apps applied as self‐management tools (e.g., due to in‐app encouragements and advice on ways to recover) (Nitsch et al., 2016, 2019).

The most significant factor predicting changes in patient app use in our study was the passing of time, which could be explained by changes in symptoms. Patients’ condition may have improved markedly making them less inclined to log as much as initially due to a decreased need for daily assistance to eat regularly (Lindgreen, Lomborg, et al., 2018). Oppositely, patients’ symptoms may have worsened and become a barrier to logging. In some cases, logging or reviewing meal logs may induce feelings of guilt when revisiting the amount of food eaten, which might ensue worsened symptoms (Lindgreen, Lomborg et al., 2018) . Missing data on changes in symptom severity levels, however, makes it impossible for us to know to what degree these factors affected app engagement.

Clinicians’ use of app data in session may also have affected patients’ app engagement; patients may have felt disappointed and demotivated if their clinician did not actively incorporate their app data as part of treatment sessions. In such cases, logging continuously may have felt like a waste of time and energy for patients (Lindgreen, Lomborg, et al., 2018). According to previous research, a lack of clinician use of app data may have occurred, as clinicians have reported the amount of app data to be overwhelming and found it unrealistic to review in the short period of time available to prepare each session, during which other competing tasks were in need of solving as well (e.g., conferring with colleagues and documenting events in medical records) (Lindgreen, Clausen, et al., 2018). Thus, clinicians may have prioritized other aspects of treatment over the appraisal of patient app data. Other clinicians simply have chosen not to review patients’ logs before sessions to maintain an “open mind” and let the patients report on their progress themselves (Lindgreen, Clausen, et al., 2018). To avoid patient disappointment and clinicians feeling overwhelmed by the amount of app data, clinical guidelines on the use of treatment apps are needed to accommodate the needs and preferences of both parties (Lindgreen, Clausen et al., 2018 ). Such guidelines should include key points for clinicians and patients to discuss to ensure that expectations are aligned regarding the degree to which patient app data is reviewed in advance of and applied in sessions ( Lindgreen, Clausen, et al., 2018). Preferably, the guidelines should be developed, implemented, and adjusted both before and during app use, which may be a challenges, as several clinics tend to implement apps prematurely out of eagerness to apply novel technologies (Fairburn & Rothwell, 2015).

Previous ED treatment predicted a lower number of “active” app weeks, which may suggest that prolonged and persistent EDs limit patients’ app activity. This is in line with other studies finding that previous treatment and severe symptoms are associated with less positive outcomes (Cooper et al., 2016; Halmi, 2013), which can be mediated by readiness to change (Bewell & Carter, 2008). Thus, lower app activity in patients with previous courses of ED treatment might be linked to reduced motivation levels to engage in behavior change, including self‐monitoring. No matter the reason, clinicians should be aware that patients may lack the motivation to engage in an app long‐term. In these cases, the clinical relevance of using a self‐monitoring app should be discussed, and the patient's need for additional support to self‐monitor should be considered. Future longitudinal studies on long‐term patient app engagement might benefit from the inclusion of patient motivation measures at baseline and throughout the study period. Otherwise, patients might become disappointed in themselves and further demotivated to recover if failing to comply with treatment recommendations (Denison‐Day et al., 2018), including long‐term self‐monitoring.

We found older age to be associated with higher app activity, whereas younger patients were less active. We expected the opposite, as younger people are usually more comfortable and familiar with smartphone apps (Donovan, 2016). Our finding might indicate that this is not the case; that being familiar with apps in general is not predictive of patient engagement in treatment‐specific apps; or that age was not an expression of app familiarity. Previous research has found adolescent patients in ED treatment to be increasingly motivated to recover with age (Zaitsoff & Taylor, 2009). Our finding may therefore illustrate that older patients logged more as they were more motivated to recover and comply with treatment recommendations (i.e., self‐monitoring). Exploring this hypothesis further highlights the need for future studies on long‐term app engagement to collect data on patients’ level of motivation to recover.

In our study, patients primarily recorded meal logs and meal photographs/thoughts/feelings logs, but rarely any of the remaining log types. Unfortunately, we cannot explain the limited patient use of RR features that include more cognitive or emotional aspects. However, one reason may be clinician recommendations, for instance if emphasizing selected log types over others, or a limited guidance of patients on how to use different app features. Thus, future trials on long‐term patient app engagement should test the potential influence of clinician recommendations and practices on patient app use by collecting data on how and to what degree clinicians use patient app data in sessions, or if patients have specific reasons for a one‐sided app use (e.g., feeling overwhelmed by the many features and tasks included in RR (Lindgreen, Lomborg, et al., 2018).

### 6.1 Strengths and limitations

To our knowledge, our study is the first to explore the use of RR in a clinical sample of ED patients for a duration of 26 weeks. Still, our study holds limitations. To optimize patients’ treatment adherence, the patients and clinicians collaborated on selecting the appropriate self‐monitoring tool (i.e., pen‐and‐paper or RR), which could, but usually did not, change during treatment. Although this nonstandardized approach complicates the interpretation of the findings, it corresponds with the aim of exploring patient app use in naturalistic ED treatment.

Unfortunately, we were unable to explore the association between app activity and ED symptoms, as the EDE‐Q data were omitted due to missing data. Yet, as the link between patients’ long‐term app engagement and their symptom development is seemingly important to explore, we recommend that future studies collect longitudinal data on patient app engagement as well as changes in ED symptoms and severity similarly to the longitudinal study on RR (Chapa et al., 2019). As we were unsuccessful collecting these data using self‐report measures, future studies may consider using clinician‐collected data when possible, such as the EDE (Fairburn et al., 2008).

The large confidence intervals for the total number of logs in our study suggest a lack of statistical power, possibly introducing type II errors. Furthermore, we applied a median split approach to define “high” and “low” app activity groups, because no commonly agreed upon definitions of app activity levels exist, and to avoid the loss of statistical power due to the small sample size (Iacobucci et al., 2015a, 2015b). However, this approach may have concealed the individual‐level variation in the data, thereby potentially reducing the quality of the results. Still, the main outcome variable of number of logs was kept continuous in the regression analyses, in which no significant differences were found between diagnoses.

To our knowledge, no commonly agreed upon definitions of “high” versus “low” long‐term app engagement in mental health apps exist, as the content, purpose, and tasks of the various apps available may vary greatly (Ng et al., 2019) which led us to apply our own categories based on patients’ grouped daily app activity levels in terms of number of logs. We recommend that future studies consider other methods of exploring app engagement that account for intraindividual‐level long‐term app engagement changes to a greater extend, such as individual‐level time series modeling (Molenaar et al., 2009). Researchers should also consider other indicators of app engagement than merely the number of logs performed, such as the number of minutes spent daily on the app (Boers et al., 2019).

Another limitation is the lack of data on the clinicians’ use of RR before and during treatment sessions, which should be collected in future studies.

Lastly, the only measure of interpersonal functioning in our study was relationship status, which may not be a sufficient measure of interpersonal functioning and the impact of social networks. Thus, future trials may benefit from the collection of data on the availability of various social networks (e.g., partner, family, friends, coworkers, and social media groups) and the degree to which they are providing the desired social support in general and in terms of ED treatment and app engagement specifically (Geller et al., 1990, 2017). We did not find interpersonal functioning to affect app engagement. However, we cannot exclude the fact that other and more concise measures of interpersonal functioning, such as the level of experienced social support, may detect a potential impact on app engagement.

Patient app use varied greatly, the number of logs rapidly decreased, and several log types were not employed. Based on the findings of this study, it is unlikely that ED diagnoses substantially influence patients’ long‐term app engagement, but age, previous ED treatment, and time may be relevant predictors. Future research ought to explore the impact of patient treatment motivation on their app activity, and the link between app activity and ED symptom changes over time.

## CONFLICT OF INTERESTS

In 2016, the first author completed a 6‐week research stay with Recovery Record Inc. who has developed the investigated app. Still, neither Recovery Record Inc. nor the funding parties have influenced the study design, analyses, results, or manuscript. The authors have no other conflicts to declare.

## AUTHOR CONTRIBUTIONS

All three authors contributed substantially to the conceptualization, design, data curation, and analyses conducted in the study as well as the writing, editing, and reviewing of the manuscript.

### Peer Review

The peer review history for this article is available at https://publons.com/publon/10.1002/brb3.2039.

## Data Availability

The data that support the findings of this study are available on request from the corresponding author. The data are not publicly available due to privacy or ethical restrictions.
